# Gitelman syndrome

**DOI:** 10.1186/1750-1172-3-22

**Published:** 2008-07-30

**Authors:** Nine VAM Knoers, Elena N Levtchenko

**Affiliations:** 1 Department of Human Genetics, Radboud University Nijmegen Medical Centre, Nijmegen, The Netherlands

## Abstract

Gitelman syndrome (GS), also referred to as familial hypokalemia-hypomagnesemia, is characterized by hypokalemic metabolic alkalosis in combination with significant hypomagnesemia and low urinary calcium excretion. The prevalence is estimated at approximately 1:40,000 and accordingly, the prevalence of heterozygotes is approximately 1% in Caucasian populations, making it one of the most frequent inherited renal tubular disorders. In the majority of cases, symptoms do not appear before the age of six years and the disease is usually diagnosed during adolescence or adulthood. Transient periods of muscle weakness and tetany, sometimes accompanied by abdominal pain, vomiting and fever are often seen in GS patients. Paresthesias, especially in the face, frequently occur. Remarkably, some patients are completely asymptomatic except for the appearance at adult age of chondrocalcinosis that causes swelling, local heat, and tenderness over the affected joints. Blood pressure is lower than that in the general population. Sudden cardiac arrest has been reported occasionally. In general, growth is normal but can be delayed in those GS patients with severe hypokalemia and hypomagnesemia.

GS is transmitted as an autosomal recessive trait. Mutations in the solute carrier family12, member 3 gene, *SLC12A3*, which encodes the thiazide-sensitive NaCl cotransporter (NCC), are found in the majority of GS patients. At present, more than 140 different NCC mutations throughout the whole protein have been identified. In a small minority of GS patients, mutations in the *CLCNKB *gene, encoding the chloride channel ClC-Kb have been identified.

Diagnosis is based on the clinical symptoms and biochemical abnormalities (hypokalemia, metabolic alkalosis, hypomagnesemia and hypocalciuria). Bartter syndrome (especially type III) is the most important genetic disorder to consider in the differential diagnosis of GS. Genetic counseling is important. Antenatal diagnosis for GS is technically feasible but not advised because of the good prognosis in the majority of patients.

Most asymptomatic patients with GS remain untreated and undergo ambulatory monitoring, once a year, generally by nephrologists. Lifelong supplementation of magnesium (magnesium-oxide and magnesium-sulfate) is recommended. Cardiac work-up should be offered to screen for risk factors of cardiac arrhythmias. All GS patients are encouraged to maintain a high-sodium and high potassium diet. In general, the long-term prognosis of GS is excellent.

## Disease name and synonyms

Gitelman syndrome

Gitelman's syndrome

Familial hypokalemia-hypomagnesemia

## Definition and epidemiology

Gitelman syndrome (GS) (OMIM 263800), also referred to as familial hypokalemia-hypomagnesemia, is an autosomal recessive salt-losing renal tubulopathy that is characterized by hypomagnesemia, hypocalciuria and secondary aldosteronism, which is responsible for hypokalemia and metabolic alkalosis [1]. The prevalence is estimated at ~25 per million and accordingly, the prevalence of heterozygotes is approximately 1% in Caucasian populations, making it one of the most frequent inherited renal tubular disorders.

## Clinical description

GS patients usually present above six years of age and in many cases the diagnosis is only made at adult age. Most patients suffer from tetany, especially during periods of fever or when extra magnesium is lost due to vomiting or diarrhea. Paresthesias, especially in the face, frequently occur. Some patients experience severe fatigue interfering with daily activities, while others never complain of tiredness. The severity of fatigue in GS is not completely related to the degree of hypokalemia. In contrast to Bartter syndrome (a genetically distinct and clinically more severe tubular transport disorder, which shares the hypokalemic metabolic alkalosis with GS) polyuria is usually absent or only mild. In general, growth is normal in GS patients, however, it can be delayed in patients with severe hypokalemia and hypomagnesemia [2].

Some adult GS patients suffer from chondrocalcinosis, which is assumed to result from chronic hypomagnesemia. It causes swelling, local heat, and tenderness over the affected joints. In earlier clinical reports additional symptoms, such as ataxia, vertigo, and blurred vision have been reported.

Cruz and colleagues have challenged the generally shared idea that GS is a mild disorder [3]. They evaluated the symptoms and quality of life (QOL) in 50 adult patients with molecularly proven GS and compared this cohort of patients with 25 age- and sex-matched controls. They found that GS patients had significantly more complaints than controls, mainly salt craving, musculoskeletal symptoms such as tetany and cramps, muscle weakness and aches, and constitutional symptoms such as fatigue, generalized weakness and dizziness, and nocturia and polydipsia. In addition, measures of QOL were significantly lower in GS patients compared to controls.

Potassium and magnesium depletion prolong the duration of the action potential of cardiomyocytes and consequently increase the risk for development of ventricular arrhythmia. Electrocardiograms of patients with Gitelman syndrome have shown that in about 50% of cases the QT interval is indeed slightly to moderately prolonged but, fortunately, is not associated with clinically relevant cardiac arrhythmias in the far majority of cases [4]. Sudden cardiac arrest reported in few patients with GS [2,5], warrants systematic cardiac screening for identifying other possible triggering mechanisms or underlying conditions. Blood pressure in GS patients is lower than in the general population, indicating that even the modest salt wasting of this disease reduces blood pressure. The results from a recent study in 35 GS-carriers (with one mutant gene allele) suggest that GS carriers also have lower blood pressure and may be protected from hypertension [6]. Another study in a large cohort also demonstrated reduced blood pressure in subjects having *SLC12A3 *mutations on one allele [7]. These results are distinct from a previous study in an Amish kindred, in which no reduction of blood pressure was demonstrated in adult patients, despite increased Na^+ ^excretion [8]. Thus, further studies are required to investigate whether the incidence of cardio-vascular events differs between GS patients or carries compared to control population.

## Etiopathogenesis

In the great majority of cases GS is caused by mutations in the solute carrier family 12, member 3, *SLC12A3 *gene, which encodes the renal thiazide-sensitive sodium-chloride co-transporter NCC that is specifically expressed in the apical membrane of cells in the first part of the distal convoluted tubule (DCT) (reviewed in [9]). NaCl cotransporter (NCC) is a polypeptide of 1021 amino acids and the 2D-structure is predicted to contain 12 transmembrane domains and long intracellular amino- and carboxytermini. At present, more than 140 different, putative loss-of-function mutations in the *SLC12A3 *gene have been identified in GS patients. These mutations include missense-, nonsense-, frame-shift-, and splice-site mutations and are distributed throughout the whole protein.

In general, there is extreme inter- and intrafamiliar phenotype variability in GS, the latter emphasizing the lack of a correlation between the severity of symptoms in GS and the type of mutation in the *SLC12A3 *gene [10]. Recently, however, Riviera-Munoz *et al*. described a small subgroup of patients with a remarkable severe phenotype, including an early onset, severe neuromuscular manifestations, growth retardation and ventricular arrhythmias [2]. The majority of these patients were male and carried at least one allele of a splice defect, resulting in a truncating transcript, or a non-functional intracellularly retained mutation (see below). They suggested from these data that the nature/position of the *SLC12A3 *mutation combined with male gender may be a determinant factor in the severity of GS. Studies in lager cohorts are necessary to confirm this assumption.

By functional expression studies and results of immunocytochemistry in *Xenopus leavis *oocytes, it was shown that most disease-causing NCC mutants are impaired in their routing to the plasma membrane. Thus, the majority of mutations belong to the so-called type 2 mutations which, in contrast to type 1 mutations that impair protein synthesis, lead to fully synthesized proteins. These type 2 mutant proteins, however, do not traffic appropriately to the plasma membrane, primarily due to protein misfolding and retention in the endoplasmic reticulum, followed by rapid proteoasomal degradation. De Jong and co-workers have shown that NCC misfolding resulting in defective trafficking in GS is not uniformly complete [11]. Some mutant NCC proteins are only partially retarded in their trafficking; they do reach the plasma membrane, albeit to a limited extent, and are partially active. Subsequently, it was demonstrated that the intrinsic activity of these partially retarded mutants is unaffected by the mutation [12,13]. This finding opens the possibility of pharmacological chaperones, facilitating the routing of misfolded, trafficking-defective, but otherwise functional NCCs to the apical membrane, for therapeutic use. Indeed, in an additional study, de Jong *et al. *found prove that the transcriptional regulator 4-phenylbutyrate may be a promising candidate for rescuing partially retarded, but otherwise functional mutant NCCs [14]. Recently, another class of mutations in GS was identified by Riveira-Munoz *et al. *[15]. This newly identified class includes mutants which are partly retained in the cell, but in contrast to the mutants mentioned above, these mutants do not show any activity when they reach the cell surface.

A minority of patients with the Gitelman phenotype has been shown to have mutations in the *CLCNKB *gene, encoding the renal chloride channel ClC-Kb, located in basolateral membrane of cells of the thick ascending limb of Henle's loop (TAL) and the distal tubules. Mutations in the *CLCNKB *gene were previously found to be the cause of classic Bartter syndrome. It is now evident that the clinical phenotype in patients with *CLCNKB *mutations can be highly variable, from an antenatal onset of Bartter syndrome on one side of the spectrum, to a phenotype closely resembling Gitelman syndrome at the other side (review in [9]). Therefore, there is an indication to screen the *CLCNKB *gene in patients with the Gitelman phenotype who do not have mutations in the *SLC12A3 *gene.

**Figure 1:**
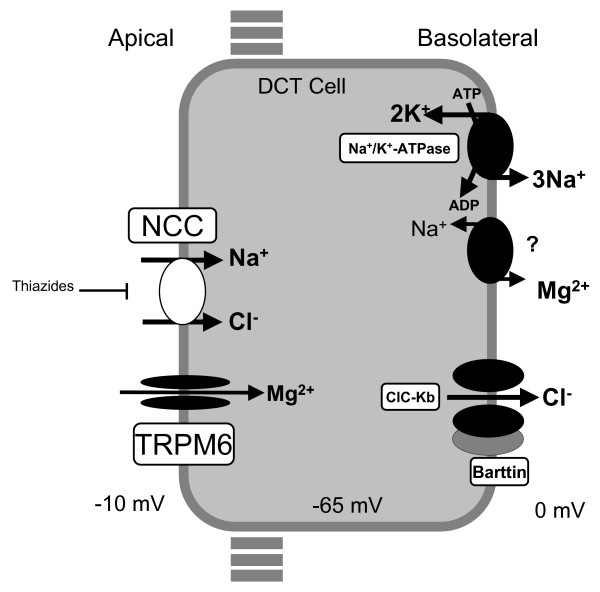
**A model of transport mechanisms in the DCT**. Sodium-chloride (NaCl) enters the cell via the apical thiazide-sensitive NCC and leaves the cell through the basolateral Cl^- ^channel *(ClC-Kb)*, and the Na^+^/K^+^-ATPase. Indicated also are the recently identified magnesium channel TRPM6 in the apical membrane, and a putative Na/Mg exchanger in the basolateral membrane.

Both loss-of-function mutations in NCC and mutations in CLC-Kb lead to disruption of NaCl reabsorption in the DCT (figure 1). When less NaCl is reabsorped, more sodium will arrive in the collecting duct resulting in mild volume contraction. The reduced vascular volume activates the renin-angiotensin-aldosterone system, increasing renin activity and aldosterone levels. The elevated aldosterone levels give rise to increased electrogenic sodium reabsorption in the cortical collecting duct *via *the epithelial sodium channel (ENaC), defending salt homeostasis at the expense of increased secretion of potassium and hydrogen ions, thus resulting in hypokalemia and metabolic alkalosis. It has been shown that passive Ca^2+ ^reabsorption in the proximal tubule and reduced abundance of the epithelial Mg^2+ ^channel TRPM6, located in the DCT explains thiazide-induced hypocalciuria and hypomagnesemia, respectively [16]. Since thiazides are known to inhibit NCC, and in view of the phenotypic resemblance between GS and chronic thiazide-treatment, it is very likely that similar mechanisms are involved in the pathogenesis of respectively hypocalciuria and hypomagnesemia seen in GS.

## Diagnosis, diagnostic methods and differential diagnosis

The diagnosis of Gitelman syndrome is based on the clinical symptoms and biochemical abnormalities. The most typical biochemical abnormalities in GS are hypokalemia, metabolic alkalosis, hypomagnesemia and hypocalciuria. Serum potassium concentration is comparably low (2.7 ± 0.4 mmol/L) to Bartter syndrome. Serum magnesium concentration is low (less than 0.65 mmol/l). In a few GS patients magnesium concentration is easily maintained in the normal range early on, which may lead to a false diagnosis of Bartter syndrome, and only drops below normal with time (personal observation). Urinary calcium concentration is usually less than 0.2 mmol/mmol creatinine and rarely exceeds 0.5 mg/kg/day. Hypomagnesemia and hypocalciuria have always been considered obligate features for GS. This assumption has recently been disputed by Lin *et al. *[10]. They reported two families with molecularly proven GS, in which male patients had severe hypokalemia, and were symptomatic with episodes of paralysis, impaired urinary concentration ability, but with normal serum magnesium and urinary calcium excretion. Remarkably, female GS patients within these families, carrying the same causative mutations as the male patients, were asymptomatic, had less severe hypokalemia, intact urine concentration ability, but did have hypomagnesemia and hypocalciuria [10]. Although this was a small study, the authors concluded that gender may affect phenotypic expression in GS and that hypomagnesemia and hypocalciuria may not be invariant features of the disorder.

Prostaglandin excretion is normal and plasma renin activity and plasma aldosterone concentration are only slightly elevated compared to Bartter syndrome. Renal functional studies have demonstrated normal or slightly decreased urinary concentrating mechanism, but clearly reduced distal fractional chloride reabsorption during hypotonic saline infusion. GS patients have a blunted natriuretic response to hydrochlorothiazide administration but a prompt natriuresis after administration of furosemide, indicating that the defect in GS is located at the level of the distal tubule. DNA mutation analysis of the gene responsible for GS may confirm the diagnosis.

Bartter syndrome is the most important genetic disorder to consider in the differential diagnosis of GS. Especially type III Bartter syndrome, which is caused by mutations in the *CLCNKB *gene, is clinically and biochemically overlapping with Gitelman syndrome. The other types of Bartter syndrome usually have an earlier onset and a more severe phenotype.

Primary forms of renal hypomagnesemia can be distinguished from GS by the absence of hypokalemia. Important acquired conditions which should be differentiated from GS are diuretic and laxative abuse and chronic vomiting. The two latter conditions can be confirmed by measuring of low urinary excretion of Cl^-^.

## Genetic counseling

Genetic counseling is important. Since GS is an autosomal recessive trait, the recurrence risk for parents with an affected child is 25%. If the parents already have other children, who are not obviously affected, it is not absolutely sure that they do not have GS because clinical symptoms can appear later in life. If the parents are eager to know the status of the other child(ren) and in case the molecular defect in their affected child has been elucidated, DNA-analysis in the other child(ren) may be performed. Adult patients with GS have a low risk of having children with GS (~1 in 400) unless the patient and his/her partner are consanguineous. Although technically feasible, antenatal diagnosis for GS is not advised and as yet has never been asked for because of the good prognosis in the majority of patients.

## Management including treatment

Most asymptomatic patients with GS remain untreated and undergo ambulatory monitoring (generally by nephrologists) with low frequency (1–2 times per year). At each visit complaints related to hypokalemia (fatigue, muscle weakness, constipation, cardiac arrhythmias) and hypomagnesemia (tetany, cramps, paresthesias, joint and muscle pain) as well as serum levels of K^+^, bicarbonate and Mg^2+ ^should be evaluated. In view of the assumption that chondrocalcinosis is due to magnesium deficiency (magnesium is a co-factor of various pyrophospatases, including alkaline phosphatase), there is a clear argument for lifelong supplementation of magnesium. Normalization of serum magnesium is difficult to achieve since high doses of magnesium cause diarrhea. The bio-availability of magnesium preparations is different. Magnesium-oxide and magnesium-sulfate have a significantly lower bio-availability compared to magnesium-chloride, magnesium-lactate and magnesium-aspartate. We recommend the administration of magnesium-chloride orally to compensate for renal Mg^2+ ^and Cl^- ^losses. Initial daily dose is 3 mmol Mg/m^2^/24 hrs or 4–5 mg/kg/24 hrs. This dose should be divided in 3–4 administrations to avoid diarrhea and has to be adjusted according to serum magnesium levels. The dose usually has to be increased during periods of undercurrent infections especially those accompanied by vomiting and diarrhea. In case of acute tetany, 20% MgCl_2 _should be administered intravenously (0.1 mmol Mg/kg per dose) and can be repeated every 6 hours.

Complaints related to chondrocalcinosis (mainly pseudogout attacks) are caused by the deposition of calcium pyrophosphate dehydrate crystals in synovium and the synovial fluid and can be reduced by Mg^2+ ^supplementation [17]. The symptoms can be controlled by non-steroidal anti-inflammatory drugs (NSAID) and joint surgery is generally not required.

If symptomatic hypokalemia is not corrected by MgCl_2 _administration, it can be treated by drugs that antagonize the activity of aldosterone or block the sodium channel ENaC in the collecting duct. We prefer the combination of amiloride (5–10 mg/1.73 m2/day) with KCl (1–3 mmol/kg/day divided in 3–4 doses). Amiloride should be started with caution in order to avoid hypotension.

Growth and puberty delay in some patients with severe GS can be corrected by adequate Mg and K supplementation and a growth-promoting effect of indomethacin was also reported in GS patients [18]. Cardiac work-up is recommended to screen for risk factors of cardiac arrhythmias. All patients with GS are encouraged to maintain a high-sodium and high potassium diet.

## Prognosis

In general, the long-term prognosis of Gitelman syndrome is excellent. However, the severity of fatigue may seriously hamper some patients in their daily activities. Progression to renal insufficiency is extremely rare in GS. As yet, only one patient who developed chronic renal insufficiency and subsequent progression to end-stage renal disease has been reported [19].

## Abbreviations

GS: Gitelman syndrome; QOL: Quality of life; DCT: Distal convoluted tubule; NCC: Thiazide-sensitive NaCl cotransporter; TAL: Thick ascending limb of Henle's loop; ENaC: Epithelial sodium channel; TRPM6: Transient receptor potential channel subfamily M, member 6; NSAID; Non-steroidal anti-inflammatory drugs.

## Competing interests

The authors declare that they have no competing interests.

## Authors' contributions

The authors contributed to this review article. They read and approved the final version of the manuscript.
